# Assembly, Characterization, and Phylogenetic Insights from the Complete Mitochondrial Genome of *Cleisthenes herzensteini* (Pleuronectiformes: Pleuronectidae)

**DOI:** 10.3390/biology15030216

**Published:** 2026-01-23

**Authors:** Guangliang Teng, Yue Miao, Yongsong Zhao, Tangyi Qian, Xiujuan Shan

**Affiliations:** 1 Key Laboratory of Sustainable Development of Marine Fisheries, Ministry of Agriculture and Rural Affairs, Yellow Sea Fisheries Research Institute, Chinese Academy of Fishery Sciences, Qingdao 266071, China; tenggl@ysfri.ac.cn (G.T.); miaoyue97@163.com (Y.M.); zhaoys@ysfri.ac.cn (Y.Z.); qiantangyi19@126.com (T.Q.); 2 Shandong Changdao Fishery Resources National Field Observation and Research Station, Yantai 265800, China; 3 Laboratory for Marine Fisheries Science and Food Production Processes, Qingdao Marine Science and Technology Center, Qingdao 266237, China

**Keywords:** *Cleisthenes herzensteini*, mitochondrial genome, phylogeny, pleuronectiformes

## Abstract

*Cleisthenes herzensteini* is a commercially important demersal fish in the Northwest Pacific. However, the resource stock of this species has undergone a drastic decline due to overfishing and habitat degradation. As a representative taxon for benthic adaptation in the order Pleuronectiformes, the molecular mechanisms underlying its specialized phenotypic traits remain poorly elucidated. Furthermore, population-level studies focusing on the mitochondrial genome of *Cleisthenes herzensteini* are currently scarce. Given that the mitochondrial genome serves as an ideal genetic tool for deciphering species evolution and population genetics, sequencing of its mitogenome will help fill critical gaps in genetic resources and provide essential support for species conservation and phylogenetic research. In this study, we sequenced, assembled, and annotated its complete mitochondrial genome. The circular mitogenome is 17,171 bp in length and exhibits a typical A + T bias (54.04%). Repeat sequence analysis identified 35 dispersed repeats. Codon usage analysis revealed that leucine was the most frequently encoded amino acid, with CUU being the preferred codon. Several protein-coding genes possessed incomplete stop codons (T--/TA-), and a nucleotide preference for A and C was observed at the third codon position. Phylogenetic reconstruction based on mitogenomes from 23 species supported the monophyly of the order Pleuronectiformes. *C. herzensteini* showed the closest relationship with *Dexistes rikuzenius*, forming a distinct clade alongside *Hippoglossoides dubius* and *Limanda aspera*. These results provide essential genetic resources for understanding the evolution and population genetics of *C. herzensteini* and related flatfishes. According to the investigation, this study represents the first report on the sequencing and analysis of the complete mitochondrial genome of the *Cleisthenes herzensteini*. This not only fills the gap in mitochondrial genetic information for this species but also provides a reference for subsequent investigations into the phylogenetic relationships and evolutionary processes within the family Pleuronectidae.

## Simple Summary

The pointhead flounder is an important fish in the Northwest Pacific, but its population has dropped sharply due to overfishing and habitat damage. To help protect this species, we need to better understand its genetic makeup. In this study, we decoded the complete set of genetic material contained in its mitochondria—the energy-producing parts of the cell—which is useful for tracking evolution and family relationships among fish. We found that its mitochondrial genetic structure is similar to that of other related flatfish, but also has some unique features, such as preferred patterns in its genetic code and certain incomplete stop signals in genes. By comparing it with other species, we confirmed its close evolutionary relationship with another flatfish, *Dexistes rikuzenius*, and clarified its place within the broader flatfish family tree. This genetic information provides a valuable resource for scientists and conservationists, supporting future efforts in species identification, population monitoring, and the development of protection strategies for this declining fishery resource.

## 1. Introduction

*Cleisthenes herzensteini* (order Pleuronectiformes, family Pleuronectidae) is a commercially important demersal fish widely distributed in the Northwest Pacific [1]. Due to its desirable flesh quality, it constitutes one of the main commercial fisheries in the Bohai and Yellow Seas [2]. However, overfishing and habitat degradation have led to a significant decline in its population and a trend towards smaller individuals [3,4]. To enhance the conservation of marine organisms, China has enforced the summer fishing moratorium system in the Yellow Sea and Bohai Sea regions since 2003, with *Cleisthenes herzensteini* designated as a key protected species. Population dynamics monitoring of *C. herzensteini* in the southern Yellow Sea, conducted by Shan et al., demonstrated that the abundance of its recruitment stock increased substantially during the summer fishing moratorium period from 2004 to 2010, in comparison with the pre-moratorium baseline [2]. Pilot programs for the artificial breeding and stock enhancement of *Cleisthenes herzensteini* have also been initiated by several research institutions. Population decline has led to reduced genetic diversity of this species due to inbreeding, which in turn impairs the adaptive capacity of its offspring. Molecular approaches enable the detection of such variations in genetic diversity via gene sequencing, thereby providing a molecular basis for ecological conservation. Environmental pollution or climate change may also drive the selection of specific genotypes, altering the genetic structure of natural populations. Molecular methodologies can identify these adaptive genes, illuminating how environmental shifts drive evolutionary trajectories. For the conservation and sustainable utilization of *C. herzensteini* resources, it is imperative to implement habitat protection and fishing regulation measures; additionally, the investigation and analysis of population genetic structure are of equal importance [5]. Additionally, the order Pleuronectiformes represents a highly morphologically specialized group within teleost fishes, characterized by its extensive species diversity. Following mitochondrial genome assembly of *Eopsetta jordani*, Patil et al. conducted evolutionary analyses revealing that this species forms a monophyletic clade distinct from its conspecific *Eopsetta grigorjewi*. Furthermore, it exhibits significant evolutionary divergence from other species within the same family, underscoring its unique evolutionary characteristics [6]. Yang et al. similarly elucidated the phylogenetic relationships and taxonomic status of the mitochondrial genome of *Acanthopsetta nadeshnyi* [7]. Tan et al. provided a relatively comprehensive report on the structural characteristics of mitochondrial genomes from 111 species of Pleuronectiformes [3]. However, to date, reports on the mechanistic links between mitochondrial genomic structural variations and benthic adaptive evolution in *Cleisthenes herzensteini* remain scarce. Investigating its population genetic structure is therefore crucial for conservation and sustainable management [5].

The mitochondrial genome is a powerful molecular marker for phylogenetic and population genetic studies due to its maternal inheritance, relatively high mutation rate, and lack of recombination [8,9,10]. In fish, complete mitogenome sequences provide more robust phylogenetic signals compared to individual genes such as *cox1* or *cyt b* [3,11,12]. Through the sequencing and analysis of the mitochondrial genome of *Niphon spinosus*, Patil et al. clarified its phylogenetic position within the suborder Perciformes and corroborated its taxonomic status as the sole representative species of the family Percidae, thereby laying a solid foundation for subsequent conservation biology research [13]. Marnis et al. conducted mitochondrial genome sequencing and analysis of the endangered *Melanotaenia fasinensis*, providing crucial data for the genetic diversity conservation of this Indonesian endemic species and resolving its taxonomic controversies [14]. For the morphologically diverse order Pleuronectiformes, mitogenomic data have been instrumental in resolving evolutionary relationships [3,15]. Although the mitochondrial genome of *C. herzensteini* was previously reported as a brief announcement [16], a comprehensive analysis of its genome structure, repetitive elements, codon usage, and phylogenetic placement within a broader taxonomic context remains lacking.

In this study, we sequenced and assembled the complete mitogenome of *C. herzensteini* using high-throughput sequencing. We conducted detailed analyses of its structural features, nucleotide composition, repetitive sequences, and codon usage bias. Furthermore, we reconstructed a phylogenetic tree of Pleuronectiformes based on complete mitogenomes to elucidate the evolutionary position of *C. herzensteini*. Our work provides an essential genomic resource for future studies on molecular identification, population genetics, and adaptive evolution of this declining fishery species.

## 2. Materials and Methods

### 2.1. Sample Collection and DNA Sequencing

A specimen of *C. herzensteini* was collected during a routine scientific bottom-trawl survey in the Yellow Sea (36.56° N, 122.49° E). As an inherent outcome of this survey method, all captured organisms, including the specimen used in this study, were confirmed deceased upon retrieval by trained research personnel qualified in ethical assessment procedures. Muscle tissue was preserved in 95% ethanol and stored at −20 °C until use. Genomic DNA was extracted using the cetyltrimethylammonium bromide (CTAB) method. The extracted DNA was fragmented by ultrasonication. A sequencing library was constructed through end repair, adenylation, adapter ligation, and PCR amplification following the manufacturer’s protocol. Library quality was assessed, and qualified libraries were sequenced on an Illumina NovaSeq 6000 platform (Tgene Biotech Co., Ltd., Shanghai, China) to generate 150 bp paired-end reads. After quality trimming, a total of 17,163,180 clean reads (approximately 5.15 Gb of data) were obtained, with a GC content of 42.49% and high-quality bases (Q20 and Q30) accounting for 97.04% and 92.42%, respectively.

### 2.2. Mitochondrial Genome Assembly and Annotation

Raw reads were quality-trimmed using fastp (v0.23.4). To enrich mitochondrial-derived sequences, clean reads were aligned against a custom mitochondrial genome database using Bowtie2 (v2.2.4). The mitogenome was assembled using SPAdes (v3.10.1) [17] and compared to a reference sequence (NCBI accession NC_028021.1) for verification. Assembly quality was validated by mapping clean reads back to the assembled genome, which yielded an average coverage depth of approximately 1403× from 76,384 mapped read pairs. Annotation was performed using the MITOS2 (v2.1.8) [18], followed by manual curation by comparison with closely related species. The mitogenome map was visualized with OGDRAW (v1.3.1).

### 2.3. Repeat Sequence Analysis

Dispersed repeats were identified using *Vmatch* (v2.3.0) with a minimum length of 20 bp and a Hamming distance of 3. Forward, palindromic, reverse, and complementary repeats were detected.

### 2.4. KA/KS Analysis

Download mitochondrial genome sequences of other required species from NCBI. Use PhyloSuite (v1.2.3) for sequence extraction and MAFFT (v7.427) for alignment of these genes. Subsequently, calculate the Ka/Ks ratio for each gene using the MLWL method with KaKs_Calculator (v2.0). Visualize the data using boxplots generated with the ggplot2 package in R (v4.2.1) [19].

### 2.5. Codon Preference Analysis

Codon usage bias for the 13 protein-coding genes (PCGs) was assessed by calculating the Relative Synonymous Codon Usage (RSCU) using a custom Perl script.

### 2.6. Nucleotide Composition Skew Analysis

Strand asymmetry was evaluated using AT-skew [(A − T)/(A + T)] and GC-skew [(G − C)/(G + C)] [20] for the entire mitogenome and its functional regions.

### 2.7. Phylogenetic Analysis

Complete mitogenome sequences for 22 flatfish species (including *C. herzensteini* from this study) and one outgroup (*Squalus acanthias*) were downloaded from GenBank (Table 1). The 13 PCGs were concatenated and aligned using MUSCLE in MEGA11 [6]. The best-fit substitution model (GTR + G + I) was selected using the ModelFinder function in IQ-TREE (v2.4.0). A maximum-likelihood tree was constructed with 1000 bootstrap replicates. The Bayesian phylogenetic tree was constructed using MrBayes (v3.2.7), with the optimal substitution model (GTR + I + G) selected based on the Bayesian Information Criterion (BIC). Subsequently, the phylogenetic tree was visualized using the online tool iTOL (https://itol.embl.de/itol_account.cgi (accessed on 16 June 2025)).

## 3. Results

### 3.1. Structure and Composition of the Mitochondrial Genome

**Figure 1:**
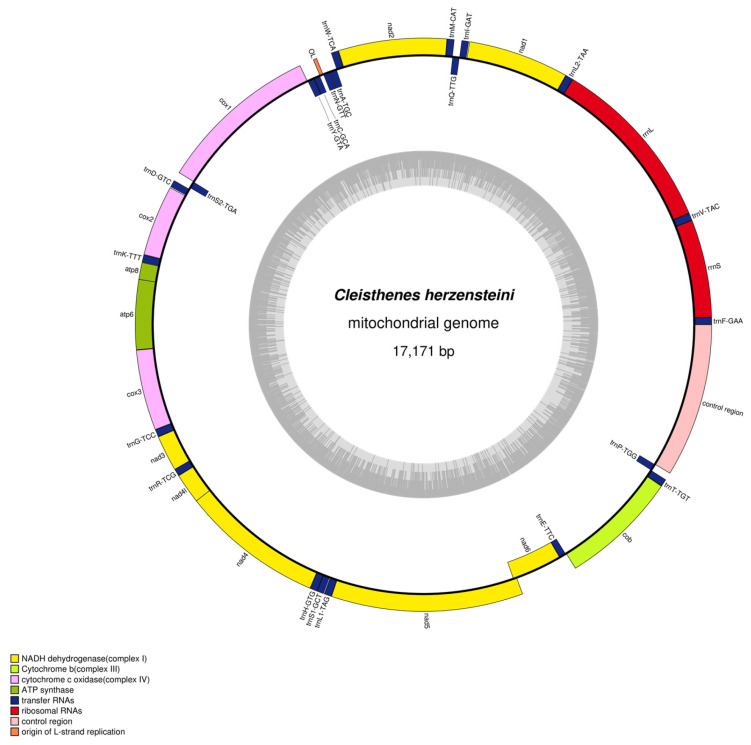
Mitochondrial Genome Map of the *C. herzensteini*.

The complete mitochondrial genome of *C. herzensteini* is a circular double-stranded DNA molecule of 17,171 bp (Figure 1). It contains the typical set of 37 genes (13 protein-coding genes (PCGs), 22 tRNA genes, and 2 rRNA genes) as well as two major non-coding regions (the origin of light-strand replication (OL) and the control region (D-loop)). Gene order was identical to that of other vertebrate mitogenomes. Eight tRNAs (*trnQ*, *trnP*, *trnE*, *trnS2*, *trnY*, *trnC*, *trnN*, and *trnA*) and the *nad6* gene were encoded on the light strand, while all other genes were located on the heavy strand (Table 2).

The overall nucleotide composition exhibited a bias toward A and T: A (27.6%), T (26.45%), G (17.13%), and C (28.82%). The A + T content (54.04%) was higher than the G + C content (45.96%). Skew analysis revealed a slightly positive AT-skew (0.021) and a negative GC-skew (−0.254) (Table 3).

### 3.2. Analysis of Dispersed Repeat Sequences

**Figure 2:**
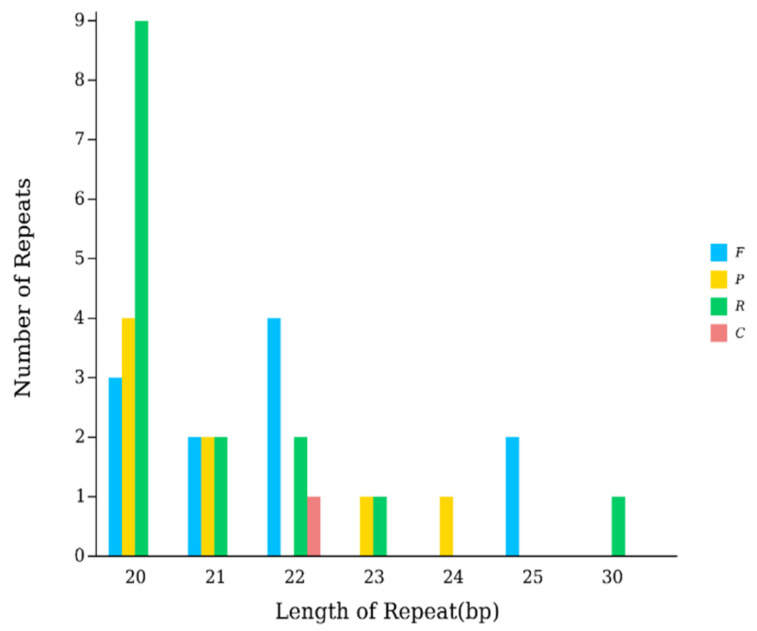
Scattered Repeat Sequences in the Mitochondrial Genome of *C. herzensteini*.

A total of 35 dispersed repeats were identified in the mitogenome. These included 8 palindromic (P), 15 inverted (R), 11 forward (F), and 1 complementary (C) repeat. Most repeats were short, with lengths ranging from 20 to 30 bp; no long repetitive sequences were detected (Figure 2).

The X-axis represents the type of scattered repeats, while the Y-axis indicates the number of scattered repeats.

### 3.3. Non-Synonymous/Synonymous Mutation Ratio (Ka/Ks) Analysis

**Figure 3:**
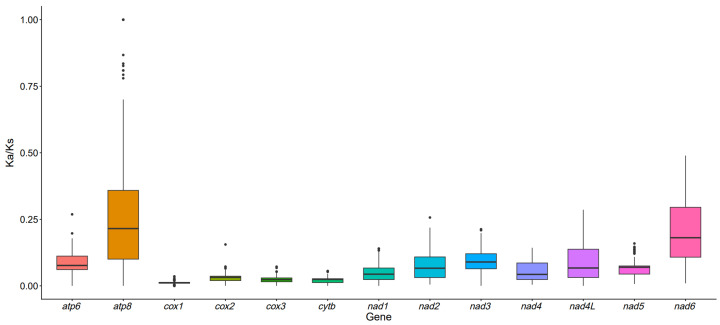
Ka/Ks analysis of PCGs in mitochondrial genomes of 23 species. Solid black lines on the box plot represent mean values.

To investigate the impact of selective pressures on mitochondrial genome evolution in the *Cleisthenes herzensteini*, the ka/Ks ratios of 13 homologous PCGs were compared between the *Cleisthenes herzensteini* and 22 other species. As shown in Figure 3, most PCGs exhibited relatively low ka/Ks values concentrated between 0 and 0.25, indicating these genes underwent purifying selection during evolution. Individual genes such as *atp8* and *nad6* exhibit higher and more dispersed values, suggesting weaker purifying selection or greater adaptive variation. Genes including *cox1, cox2, cox3*, and *cytb* exhibit extremely low ka/ks values approaching zero, indicating strong purifying selection pressure and relatively conserved sequences. *nad1*–*nad5* and *nad4L* generally show smaller ka/ks values, though variations exist among these genes, suggesting differing degrees of selective pressure.

### 3.4. Protein-Coding Genes and Codon Usage

The 13 PCGs totaled 11,448 bp in length. The start codon ATG was used by 12 PCGs, while *cox1* used GTG. Seven PCGs (*nad1*, *cox1*, *atp8*, *atp6*, *nad4l*, *nad5*, and *nad6*) used TAA as the stop codon, and *nad3* used TAG. Five genes (*nad2*, *cox2*, *nad4*, *cob*, and *cox3*) possessed incomplete stop codons (T-- or TA-) (Table 2).

**Figure 4:**
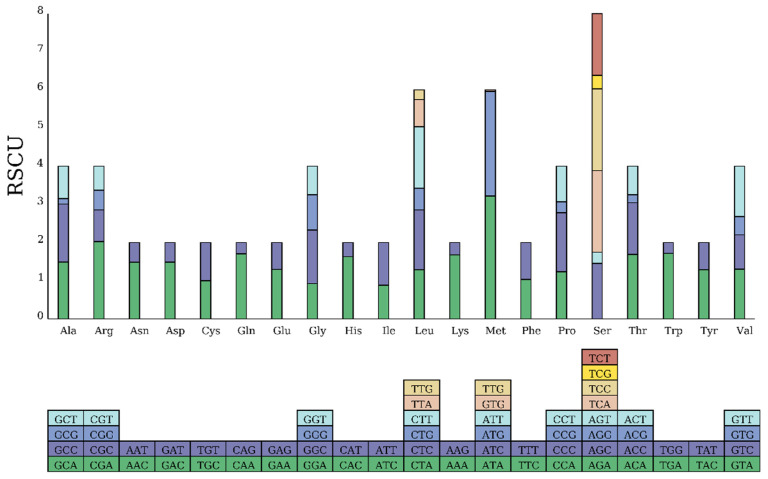
Relative synonymous codon usage in the mitochondrial genome of *C. herzensteini*.

A total of 3819 codons were analyzed. Leucine (Leu, 641 occurrences) was the most frequent amino acid, followed by alanine (Ala, 359) and threonine (Thr, 289). Cysteine (Cys, 24) was the least frequent. Relative Synonymous Codon Usage (RSCU) analysis indicated a strong preference for the codon CUU (RSCU = 1.61) for Leu. Overall, there was a marked preference for A and C at the third codon position in the PCGs (Table 4, Figure 4).

### 3.5. Ribosomal RNA and Transfer RNA Genes

The two rRNA genes (*rrnL* and *rrnS*) had a combined length of 2664 bp, accounting for 15.51% of the mitogenome. Their nucleotide composition showed an A + T content of 52.59% and a positive AT-skew (0.203) (Table 3).

**Figure 5:**
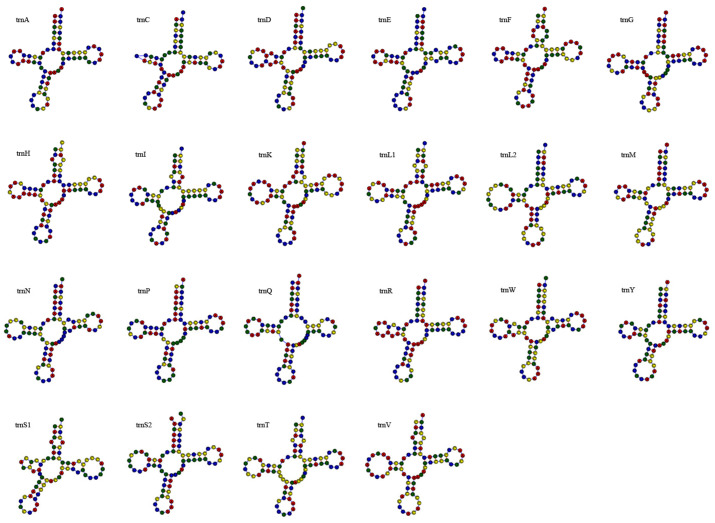
Secondary structure of 22 tRNA genes in the mitochondrial genome of *C. herzensteini*.

All 22 standard tRNA genes were identified, with lengths ranging from 65 to 74 bp. Their collective length was 1551 bp (9.03% of the genome). The tRNAs had an A + T content of 54.03% with a slight positive bias for both A and G (AT-skew = 0.026, GC-skew = 0.035) (Table 3). The secondary structure of tRNA is shown in Figure 5. All tRNA genes exhibit the typical cloverleaf secondary structure. Among the 22 tRNAs, 35 base pairs with G-U mismatches were observed in 16 tRNAs. These mismatched bases occurred in the amino acid acceptor arm (14 pairs), TΨC arm (6 pairs), anticodon arm (7 pairs), and DHU arm (8 pairs).

### 3.6. Non-Coding Control Region

The control region (D-loop) was 1494 bp long, located between *trnP* and *trnF*. It had the highest A + T content (62.92%) among all genomic regions. The OL region was 38 bp long and situated between *trnN* and *trnC* (Table 2, Figure 1).

### 3.7. Phylogenetic Analysis

**Figure 6:**
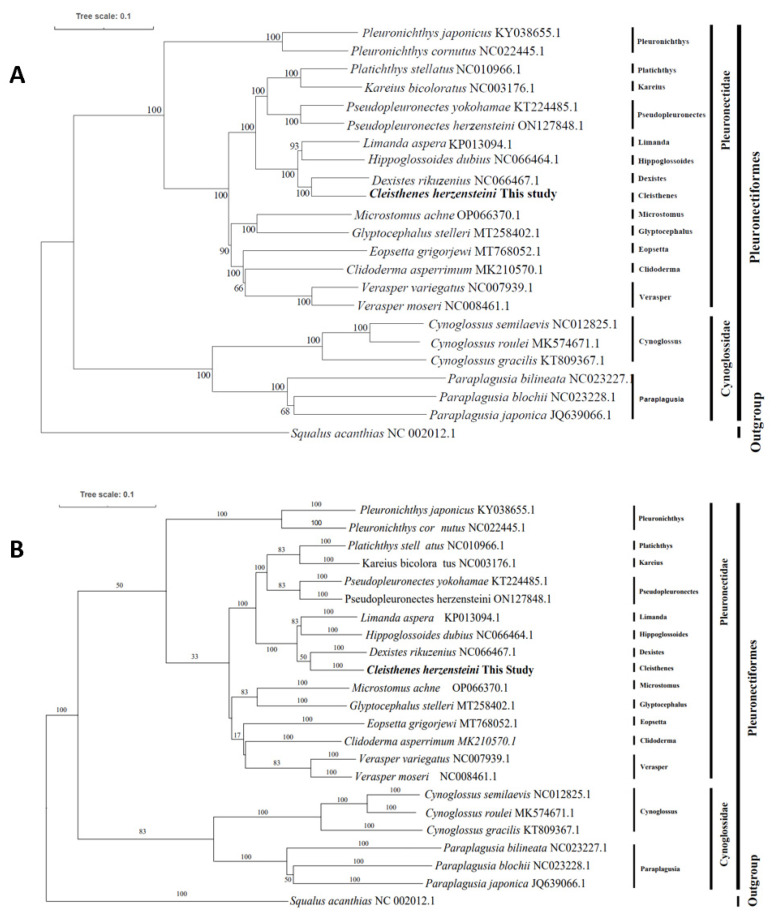
Maximum likelihood (ML) phylogenetic tree (**A**) and Bayesian inference (BI) phylogenetic tree (**B**) constructed based on the mitochondrial genomes of 23 species.

The ML and BI phylogenetic trees, constructed from the concatenated sequences of 13 PCGs from 23 species, revealed three major clades (Figure 6). All species from the family Pleuronectidae formed a monophyletic clade, which was further subdivided. *C. herzensteini* clustered most closely with *D. rikuzenius*, and together with *H. dubius* and *L. aspera*, they formed a distinct sub-clade within Pleuronectidae. Species from the family Cynoglossidae formed a separate, well-supported sister clade to Pleuronectidae. The outgroup (*S. acanthias*) was placed at the most basal position. Bootstrap values at most nodes were high, supporting the robustness of the phylogenetic relationships.

## 4. Discussion

### 4.1. Characteristics of the Mitochondrial Genome of C. herzensteini

In this study, we successfully assembled the complete mitochondrial genome of *Cleisthenes herzensteini*. Its length (17,171 bp), gene content (37 genes), and gene order are highly conserved and align with the typical pattern observed in teleost fishes and other reported flatfish species [3,21]. Furthermore, similar to other fish species, the *nad6* gene is found exclusively on the L chain. This structural conservation suggests strong functional constraints on the mitogenome organization in Pleuronectiformes [22].

**Figure 7:**
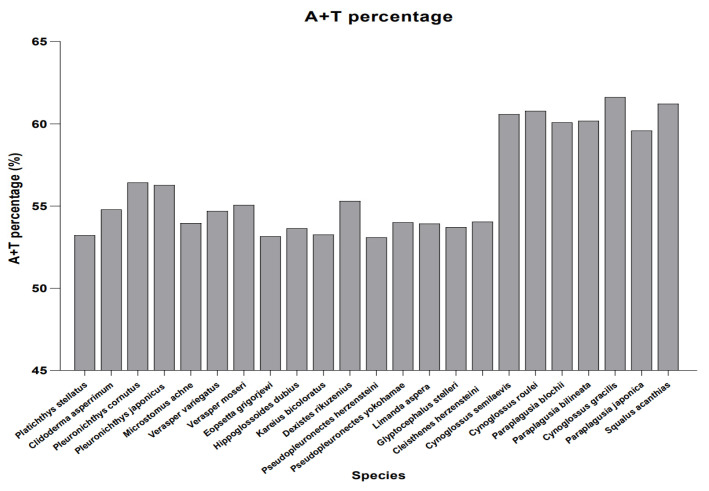
AT content percentage in mitochondrial genomes of 23 species.

The nucleotide composition of the *C. herzensteini* mitogenome exhibited a distinct A + T bias (54.04%), which is a common feature among vertebrate mitogenomes, particularly in bony fishes [23,24,25]. This bias is often attributed to asymmetric mutation pressures during replication and transcription [26]. The observed positive AT-skew and negative GC-skew are also consistent with patterns documented in many fish species, potentially reflecting strand-asymmetric replication processes [27]. A + T content, AT skew, and GC skew are three key parameters of nucleotide composition patterns. AT and GC skew vary among different species, and their evaluation can serve as reference evidence for the phylogenetic position of identified species [28]. Wei et al. found that the reversal of GC skew codes represented an independently evolved event across three distantly related insect families and was phylogenetically associated. This implies that patterns of chain asymmetry can serve as a characteristic for inferring the evolutionary history of taxonomic groups [27]. In this study, we compared the A + T content across 23 species, and the analysis revealed that the A + T content of the family Cynoglossidae is significantly higher than that of the family Pleuronectidae. This finding indicates a divergence in base composition bias between different families within the order Pleuronectiformes (Figure 7). Such a difference aligns with the taxon-specific characteristics of mitochondrial genomes and provides a base-composition-level basis for investigating the molecular evolution and taxonomic classification of Pleuronectiformes species.

Scattered repetitive sequences may participate in mitochondrial genome replication regulation by forming secondary structures. The repetitive sequences of *Cleisthenes herzensteini* are predominantly 20–30 bp in length; such short repeats are commonly distributed in non-coding regions and intergenic spacers. They share similar functions with the short repeats identified in the mitochondrial genome of *Boleophthalmus dussumieri* by Muhala et al., as they can form stem-loop structures via base complementary pairing, provide binding sites for replicases, and reduce replication errors [21]. Xiao et al. found that a lower number of repetitive sequences can reduce the genomic mutation rate and maintain the functional conservation of key genes [4].

Overlapping genes are a common feature in the mitochondrial genomes of bony fish, contributing to the compression of mitochondrial genomes and enhancing transcriptional efficiency [10,29]. However, base mutations within overlapping regions may simultaneously affect the coding products of both genes, creating evolutionary selection pressure. The sequence conservation of these overlapping regions also directly impacts the structural stability of mitochondrial DNA. We identified three short overlaps between adjacent PCGs (*nad4l*/*nad4*, *atp8*/*atp6*, and *nad5*/*nad6*), a feature contributing to genomic compaction. Similar overlapping regions are widespread in fish mitogenomes and may account for size variations among closely related species [30]. Tan et al. performed a comparative analysis of the mitochondrial genomes across 111 species of the order Pleuronectiformes and revealed that the number of gene overlaps is highly conserved within each family but exhibits significant interfamily divergence [3]. Redin et al. found in their study of the family Pleuronectidae that the conservation level of gene overlap regions in high-latitude species is significantly higher than that in low-latitude closely related species [31].

PCGs in the *Cleisthenes herzensteini* flatfish predominantly utilize the typical ATG as the start codon, while the *cox1* gene employs the GTG start codon characteristic of bony fish, consistent with previously reported findings [12,32,33]. Additionally, the use of incomplete stop codons (T-- or TA-) in five PCGs is another common phenomenon in teleosts, including other flatfish [28,34,35,36]. These incomplete codons are likely post-transcriptionally modified to complete termination signals via polyadenylation [37,38], suggesting a shared mechanism across diverse lineages. Codon usage analysis revealed a strong bias, with leucine being the most frequent amino acid and CUU as the preferred codon. A pronounced preference for A and C at the third codon position was also observed. Sebastian demonstrated in his research that the core objective of codon preference evolution is to achieve high translation efficiency and enhance mitochondrial metabolic transcription efficiency, thereby adapting to the energy demands of different habitats [39]. Such codon-usage bias is influenced by a combination of mutational pressure, natural selection for translational efficiency, and genomic nucleotide composition [40,41,42,43]. The specific pattern in *C. herzensteini* may reflect adaptive evolution related to its physiological and ecological niche.

### 4.2. Phylogenetic Relationships and Evolutionary Insights

The mitochondrial genomes follow strict maternal inheritance and undergo almost no recombination during intergenerational transmission, thus ensuring the stability of phylogenetic signals. The phylogenetic tree constructed in this study revealed a close affinity between *Cleisthenes herzensteini* and *Dexistes rikuzenius*, with a 100% bootstrap support value for the corresponding clade. This result is fully consistent with the findings of Chae et al. on the mitochondrial genome of *Microstomus achne*, confirming that maternal inheritance signals can independently and accurately reflect the generic-level phylogenetic relationships [44]. Research by Ren et al. on *Cynoglossus joyneri* also confirmed that the mitochondrial genome alone is sufficient to determine its taxonomic position within the family Cynoglossidae [45]. Our phylogenetic analysis, based on complete mitogenome data, provides strong support for the monophyly of the order Pleuronectiformes, which is consistent with some molecular studies [46,47] but remains a topic of debate when compared to morphological and other genomic evidence suggesting polyphyly. Lü et al. reported the polyphyletic origin of flatfish, with Pleuronectoidei and Psettodoidei evolving independently from their distinct Percoid ancestors [22]. Campbell et al. support the view that the flatfish phenotype has at least two independent evolutionary origins, each evolving from separate ancestors: one for *Psettodes* and one for pleuronectoids [48]. The clear separation between the families Pleuronectidae and Cynoglossidae into two distinct, well-supported clades validates their traditional family-level classification based on morphological traits [49,50].

Within the family Pleuronectidae, the phylogenetic tree revealed a complex branching pattern. *C. herzensteini* showed its closest evolutionary relationship with *D. rikuzenius*, forming a robust sub-clade that also included *H. dubius* and *L. aspera*. This grouping is novel and offers a resolved phylogenetic perspective for these genera, which had ambiguous placements in previous studies using fewer genetic markers [44,51]. Furthermore, the clustering of *Glyptocephalus stelleri* and *Microstomus achne* indicates their close evolutionary relationship, suggesting that their taxonomic status warrants further investigation using nuclear genomic data. Within the family Cynoglossidae, species form two groups that align perfectly with traditional taxonomy (based on rostrum morphology and fin ray count), corresponding to the genera *Cynoglossus* and *Paraplagusia* [52]. The extremely small genetic distance between *Cynoglossus semilaevis* and *Cynoglossus roulei* indicates they are recently diverged sister species, likely arising from ecological niche differentiation in the northwest Pacific [53]. The close relationship between *C. herzensteini* and *D. rikuzenius* may be linked to shared life-history traits or similar benthic adaptation pathways in their evolutionary history. Both the *Cleisthenes herzensteini* flounder and *Dexistes rikuzenius* prefer sandy–muddy bottom habitats and often gather in groups, which may lead to overlapping resource utilization and ecological niches. Their diets also show considerable similarity. The *Cleisthenes herzensteini* flounder primarily consumes small fish, crustaceans, and mollusks, while *Dexistes rikuzenius* also feeds on benthic organisms like crustaceans and mollusks. This dietary overlap may reflect adaptation to similar food resources, further strengthening their ecological association. Should structural variations be discovered in the mitochondrial genomes of these two species in the future, it could provide new evidence for their evolutionary relationship [54,55,56].

Furthermore, the clustering of *Glyptocephalus stelleri* with *Microstomus achne* suggests a close evolutionary link, potentially indicating that their taxonomic classification warrants further investigation with nuclear genomic data. Within Cynoglossidae, the species formed two distinct groups corresponding to the genera *Cynoglossus* and *Paraplagusia*, which aligns perfectly with traditional taxonomy based on morphological characteristics such as rostral morphology and fin ray counts [52]. The minimal genetic distance between *Cynoglossus semilaevis* and *Cynoglossus roulei* suggests they are recently diverged sister species, possibly differentiated due to ecological niche partitioning in the Northwest Pacific 53].

### 4.3. Implications and Future Perspectives

The assembled mitogenome of *C. herzensteini* serves as a fundamental genetic resource. It enables the development of species-specific molecular markers for accurate population identification, stock assessment, and monitoring of genetic diversity—all critical for the conservation and sustainable management of this declining fishery resource [2,3,5].

While mitochondrial data provide valuable insights, the evolutionary history of flatfishes is complex. Future studies integrating nuclear genomic data, expanded taxonomic sampling, and comparative genomic analyses will be essential to confirm the phylogenetic relationships proposed here, to date divergence events more precisely, and to uncover the genomic basis underlying the remarkable benthic adaptations that characterize the Pleuronectiformes [22,54,57].

## 5. Conclusions

This study presents the complete mitochondrial genome of the commercially and ecologically important flatfish, *C. herzensteini*. The assembled mitogenome is 17,171 bp in length and encodes the standard 37 genes, exhibiting conserved structural features shared among teleosts while revealing species-specific characteristics in nucleotide composition and codon usage. These findings conform to the general characteristics of the family Pleuronectidae, verifying the balance between the conservation of gene arrangement and adaptive variation during the evolutionary process of the order Pleuronectiformes. The identified characteristics, including dispersed repetitive sequences via repeat sequence analysis, codon usage bias toward Leu, and incomplete stop codons in certain genes, provide a typical case for further investigating the mutation patterns and natural selection pressures of teleost mitochondrial genomes.

Our phylogenetic analysis, based on complete mitogenome sequences, clarifies the evolutionary position of *C. herzensteini* within the order Pleuronectiformes. The results strongly support a close phylogenetic relationship between *C. herzensteini* and *D. rikuzenius*, as well as affirm the monophyly of the families Pleuronectidae and Cynoglossidae.

These findings provide a crucial molecular resource for future studies. The mitogenome sequence will facilitate the development of molecular markers for population genetics, species identification, and conservation efforts aimed at *C. herzensteini*. Furthermore, it contributes valuable data to the broader understanding of genomic evolution and phylogenetic relationships within the Pleuronectiformes.

## Figures and Tables

**Table 1:** Species and GenBank Accession Numbers Used for Phylogenetic Analysis.

Species	Family	Genus	Accession Number
*Platichthys stellatus*	Pleuronectidae	*Piatichthys*	NC_010966.1
*Clidoderma asperrimum*	Pleuronectidae	*Clidoderma*	MK210570.1
*Pleuronichthys cornutus*	Pleuronectidae	*Pleuronichthys*	NC_022445.1
*Pleuronichthys japonicus*	Pleuronectidae	*Pleuronichthys*	KY038655.1
*Microstomus achne*	Pleuronectidae	*Microstomus*	OP066370.1
*Verasper variegatus*	Pleuronectidae	*Verasper*	NC_007939.1
*Verasper moseri*	Pleuronectidae	*Verasper*	NC_008461.1
*Eopsetta grigorjewi*	Pleuronectidae	*Eopsetta*	MT768052.1
*Hippoglossoides dubius*	Pleuronectidae	*Hippoglossoides*	NC_066464.1
*Kareius bicoloratus*	Pleuronectidae	*Kareius*	NC_003176.1
*Dexistes rikuzenius*	Pleuronectidae	*Dexistes*	NC_066467.1
*Glyptocephalus stelleri*	Pleuronectidae	*Glyptocephalus*	MT258402
*Cleisthenes herzensteini*	Pleuronectidae	*Cleisthenes*	This study
*Pseudopleuronectes herzensteini*	Pleuronectidae	*Pseudopleuronectes*	ON127848.1
*Pseudopleuronectes yokohamae*	Pleuronectidae	*Pseudopleuronectes*	KT224485
*Limanda aspera*	Pleuronectidae	*Limanda*	KP013094.1
*Cynoglossus semilaevis*	Cynoglossidae	*Cynoglossus*	NC_012825.1
*Cynoglossus roulei*	Cynoglossidae	*Cynoglossus*	MK574671
*Cynoglossus gracilis*	Cynoglossidae	*Cynoglossus*	KT809367.1
*Paraplagusia blochii*	Cynoglossidae	*Paraplagusia*	NC_023228.1
*Paraplagusia bilineate*	Cynoglossidae	*Paraplagusia*	NC_023227.1
*Paraplagusia japonica*	Cynoglossidae	*Paraplagusia*	JQ639066.1
*Squalus acanthias*	Squalidae	*Squalus*	NC_002012.1

**Table 2:** Gene Annotation and Characteristics of Mitochondrial Genes in *C. herzensteini*.

Gene	Direction	Location	Size (bp)	Anticodon	Start codon	Stop codon	Intergenic Nucleotides
*trnF*	H	1–68	68	GAA			0
*rrnS (12S)*	H	69–1017	949				0
*trnV*	H	1018–1090	73	TAC			0
*rrnL (16S)*	H	1091–2805	1715				0
*trnL2*	H	2806–2879	74	TAA			0
*nad1*	H	2880–3854	975		ATG	TAA	0
*trnI*	H	3860–3929	70	GAT			5
*trnQ*	L	3929–3999	71	TTG			−1
*trnM*	H	3999–4067	69	CAT			−1
*nad2*	H	4068–5112	1045		ATG	T--	0
*trnW*	H	5113–5184	72	TCA			0
*trnA*	L	5186–5254	69	TGC			1
*trnN*	L	5256–5328	73	GTT			1
OL	H	5329–5366	38				0
*trnC*	L	5367–5431	65	GCA			0
*trnY*	L	5432–5499	68	GTA			0
*cox1*	H	5501–7060	1560		GTG	TAA	1
*trnS2*	L	7061–7131	71	TGA			0
*trnD*	H	7146–7216	71	GTC			14
*cox2*	H	7223–7913	691		ATG	T--	6
*trnK*	H	7914–7986	73	TTT			0
*atp8*	H	7988–8155	168		ATG	TAA	1
*atp6*	H	8146–8829	684		ATG	TAA	−10
*cox3*	H	8829–9613	785		ATG	TA-	−1
*trnG*	H	9614–9685	72	TCC			0
*nad3*	H	9686–10,036	351		ATG	TAG	0
*trnR*	H	10,035–10,103	69	TCG			−2
*nad4l*	H	10,104–10,400	297		ATG	TAA	0
*nad4*	H	10,394–11,774	1381		ATG	T--	−7
*trnH*	H	11,775–11,844	70	GTG			0
*trnS1*	H	11,845–11,911	67	GCT			0
*trnL1*	H	11,916–11,988	73	TAG			4
*nad5*	H	11,989–13,839	1851		ATG	TAA	0
*nad6*	L	13,802–14,320	519		ATG	TAA	−38
*trnE*	L	14,321–14,389	69	TTC			0
*cob*	H	14,394–15,534	1141		ATG	T--	4
*trnT*	H	15,535–15,607	73	TGT			0
*trnP*	L	15,607–15,677	71	TGG			−1
D-loop	H	15,678–17,171	1494				0

**Table 3:** Nucleotide Composition of the Mitochondrial Genome of *C. herzensteini*.

	Size (bp)	A%	T%	G%	C%	A + T%	G + C%	AT-Skew	GC-Skew
Mitogenome	17,171	27.6	26.45	17.13	28.82	54.04	45.96	0.021	−0.254
PCGs	11,448	24.62	28.59	16.9	29.88	53.21	46.79	−0.075	−0.277
tRNAs	1551	27.72	26.31	23.79	22.18	54.03	45.97	0.026	0.035
rRNAs	2664	31.64	20.95	21.58	25.83	52.59	47.41	0.203	−0.089
Dloop	1494	33.53	29.38	13.45	23.63	62.92	37.08	0.066	−0.274

**Table 4:** Codon Usage Frequency in the Mitochondrial Genome of *C. herzensteini*.

Codon	Count	RSCU	Codon	Count	RSCU	Codon	Count	RSCU
UAA(*)	12	1.85	AAG(K)	12	0.32	CGG(R)	10	0.52
UAG(*)	1	0.15	CUA(L)	138	1.29	CGU(R)	12	0.62
GCA(A)	134	1.49	CUC(L)	167	1.56	AGA(S)	0	0.00
GCC(A)	136	1.52	CUG(L)	61	0.57	AGC(S)	45	1.46
GCG(A)	13	0.14	CUU(L)	172	1.61	AGG(S)	0	0.00
GCU(A)	76	0.85	UUA(L)	76	0.71	AGU(S)	9	0.29
UGC(C)	12	1.00	UUG(L)	27	0.25	UCA(S)	66	2.14
UGU(C)	12	1.00	AUA(M)	81	3.22	UCC(S)	66	2.14
GAC(D)	58	1.49	AUC(M)	0	0.00	UCG(S)	11	0.36
GAU(D)	20	0.51	AUG(M)	69	2.74	UCU(S)	50	1.62
GAA(E)	63	1.30	AUU(M)	0	0.00	ACA(T)	122	1.69
GAG(E)	34	0.70	GUG(M)	1	0.04	ACC(T)	98	1.36
UUC(F)	124	1.03	UUG(M)	0	0.00	ACG(T)	15	0.21
UUU(F)	116	0.97	AAC(N)	89	1.48	ACU(T)	54	0.75
GGA(G)	58	0.92	AAU(N)	31	0.52	GUA(V)	77	1.31
GGC(G)	88	1.40	CCA(P)	70	1.23	GUC(V)	53	0.90
GGG(G)	58	0.92	CCC(P)	88	1.55	GUG(V)	28	0.47
GGU(G)	47	0.75	CCG(P)	16	0.28	GUU(V)	78	1.32
CAC(H)	84	1.63	CCU(P)	53	0.93	UGA(W)	103	1.72
CAU(H)	19	0.37	CAA(Q)	81	1.71	UGG(W)	17	0.28
AUC(I)	118	0.89	CAG(Q)	14	0.29	UAC(Y)	71	1.29
AUU(I)	148	1.11	CGA(R)	39	2.03	UAU(Y)	39	0.71
AAA(K)	63	1.68	CGC(R)	16	0.83			

Note: * denotes termination codons with no corresponding amino acid.

## Data Availability

The complete mitochondrial genome sequence of *Cleisthenes herzensteini* generated in this study has been deposited in the NCBI GenBank database under accession number [ID: PX904424].
